# Concatenating Microbial, Enzymatic, and Organometallic Catalysis for Integrated Conversion of Renewable Carbon Sources

**DOI:** 10.1021/jacsau.4c00511

**Published:** 2024-10-21

**Authors:** Nina Klos, Ole Osterthun, Hendrik G. Mengers, Patrick Lanzerath, William Graf von Westarp, Guiyeoul Lim, Marcel Gausmann, Jan-Dirk Küsters-Spöring, Jan Wiesenthal, Nils Guntermann, Lars Lauterbach, Andreas Jupke, Walter Leitner, Lars M. Blank, Jürgen Klankermayer, Dörte Rother

**Affiliations:** † Institute of Bio- and Geosciences 1: Biotechnology (IBG-1), Forschungszentrum Jülich GmbH, Jülich, Nordrhein-Westfalen 52428, Germany; ‡ Institute of Applied Microbiology (iAMB), Aachen Biology and Biotechnology (ABBt), RWTH Aachen University, Aachen, Nordrhein-Westfalen 52074, Germany; § Institute of Technical and Macromolecular Chemistry (ITMC), RWTH Aachen University, Aachen, Nordrhein-Westfalen 52074, Germany; ∥ Fluid Process Engineering (AVT.FVT), RWTH Aachen University, Aachen, Nordrhein-Westfalen 52074, Germany; ⊥ Institute of Bio- and Geosciences 2: Plant Science (IBG-2), Forschungszentrum Jülich GmbH, Jülich, Nordrhein-Westfalen 52428, Germany; # Max-Planck-Institute for Chemical Energy Conversion, Mülheim an der Ruhr, Nordrhein-Westfalen 45470, Germany

**Keywords:** Biocatalysis, Chemocatalysis, Enzymatic catalysis, Biohybrid fuels, Renewable carbon, Metabolic Engineering

## Abstract

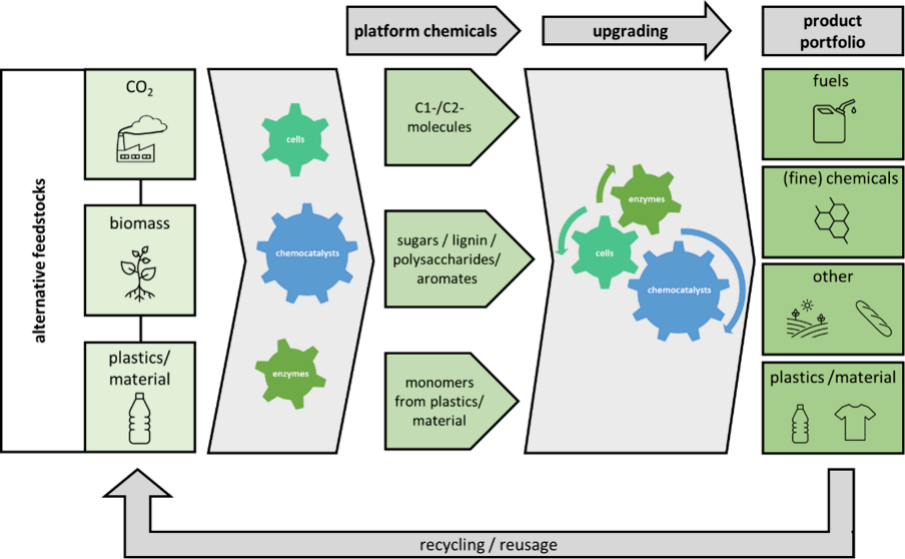

The chemical industry can now seize the opportunity to improve the sustainability of its processes by replacing fossil carbon sources with renewable alternatives such as CO_2_, biomass, and plastics, thereby thinking ahead and having a look into the future. For their conversion to intermediate and final products, different types of catalysts—microbial, enzymatic, and organometallic—can be applied. The first part of this review shows how these catalysts can work separately in parallel, each route with unique requirements and advantages. While the different types of catalysts are often seen as competitive approaches, an increasing number of examples highlight, how combinations and concatenations of catalysts of the complete spectrum can open new roads to new products. Therefore, the second part focuses on the different catalysts either in one-step, one-pot transformations or in reaction cascades. In the former, the reaction conditions must be conflated but purification steps are minimized. In the latter, each catalyst can work under optimal conditions and the “hand-over points” should be chosen according to defined criteria like minimal energy usage during separation procedures. The examples are discussed in the context of the contributions of catalysis to the envisaged (bio)economy.

## 1 Introduction

Today, chemists and biologists have a plethora of different catalysts available to target selective conversions and create value-added products. In general, chemocatalysts and biocatalysts, enable kinetically unfeasible reactions without changing the thermodynamic equilibrium and without being consumed. Nevertheless their fields of applications only rarely overlap. Most often, the different fields of applications can be grouped into the ones at physiological conditions and those at nonphysiological conditions. The former is mostly targeted by biocatalysts and the latter is targeted mostly by chemocatalysts. While the use of fossil carbon sources as feedstock for the chemical industry mostly necessitates the use of chemocatalysts, due to the nonphysiological conditions found in the refinery and intermediate product productions, the conversion of biomass can often be achieved much more selectively by the use of biocatalysts. The required substitution of fossil carbon sources with renewable carbon sources, such as carbon dioxide (CO_2_), biomass, and polymer waste, therefore, opens a window of opportunity to design transformation pathways in which chemo- and biocatalysts can work sequentially or simultaneously to achieve more selective and/or more efficient overall processes. This integration of different catalysis disciplines may enable the concatenation of chemo- and biocatalytic transformations without requiring a solvent switching (aqueous/organic) between the steps, or by designing both catalyst systems to operate together in one-pot reactions. This review gives a general overview of whole-cell catalysts (including bioelectrochemical systems), enzymes, and chemocatalysts, followed by a discussion of the various possibilities to convert renewable carbon sources and ultimately showcasing examples of exploiting the advantages of different catalysis disciplines by concatenation.

## 2 Characteristics of Catalyst Types and Challenges for Concatenation

**Figure 1:**
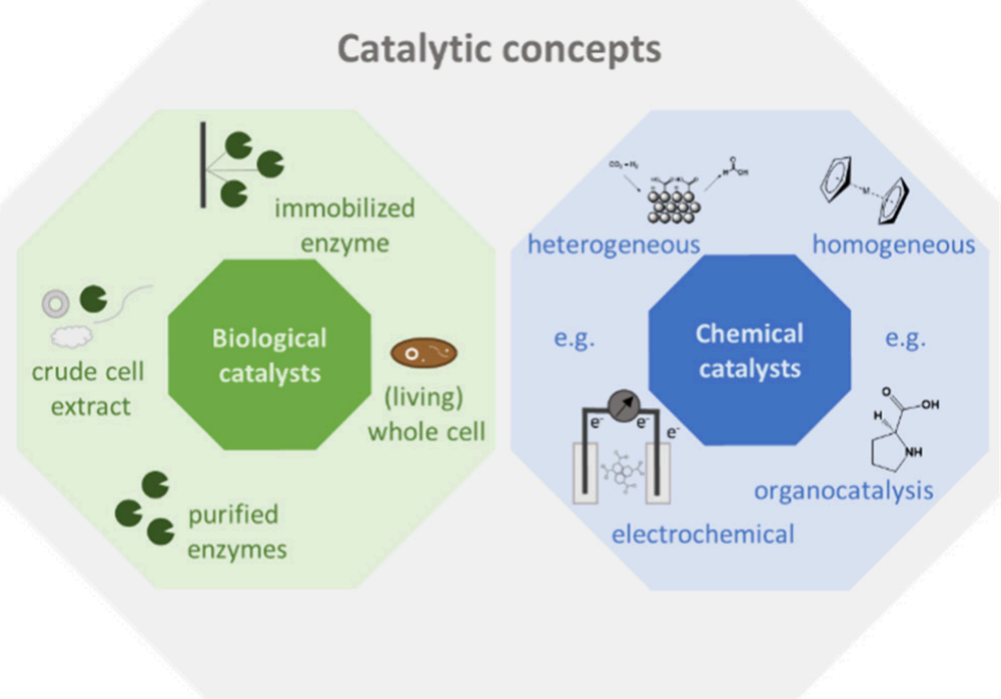
Various catalytic concepts.

There are two major areas of catalysts: biological and chemical catalysts, which are illustrated in more detail in Figure 1. Application of bacteria and yeast as biological living whole-cell catalysts, also known as microbial cell factories, is often bound to aqueous reaction media and moderate temperatures. This inherently meets many of the criteria of green chemistry.^1,2^ However, some microorganisms accept conditions such as high pressures (>1000 bar),^3^ high temperatures (up to 120 °C), acidic and basic media (pH 1 to 12), organic solvents (e.g., styrene, toluene),^4^ and high saline concentrations.^5^ The combination of extreme environments, however, is a challenge. Some microorganisms can use C1 compounds like methanol or syngas while others can degrade complex polymer materials like biological lignin or synthetic PET, although the latter is too slow for application.^6,7^ The challenge is that these single-cell specialists are mostly not (yet) accessible by genetic engineering, have therefore a very limited product spectrum, and are often not robust enough for industrial application.^8^ For this reason, most of the biotechnological production is carried out by a few heavily studied model organisms like *Escherichia coli*, *Bacillus subtilis*, or *Saccharomyces cerevisiae*.^9^ A key benefit of utilizing whole-cell catalysts is their capacity to operate as a biofactory, facilitating the simultaneous catalysis of numerous reaction steps by a single microorganism. For example, the “simple” production of bioethanol from glucose needs an entire enzymatically catalyzed metabolic pathway with more than 10 catalytic steps.^10^

In addition, enzymes can be used instead of living cells, if the targeted reaction cascade is short. Like living whole cells, the natural environment of enzymes is mostly an aqueous one and they are optimized to physiological reaction conditions. Nevertheless, enzymes can function, if carefully designed, in a continuous process, at high temperatures^11^ and in unconventional media, like microaqueous reaction systems (MARS).^12,13^ Recent advancements in enzyme engineering and reaction optimization have expanded the possibilities for utilizing harsher reaction conditions, resulting in the ability to achieve product concentrations typically associated with chemical reactions. This development has contributed to a rapid increase in applications.^14^ There are multiple ways to formulate biocatalysts: the simplest method is (lyophilized) cells, where multiple enzymes remain intact within the cell envelope. This is especially cost-effective and increases the robustness of enzymes especially for use in unconventional media such as organic solvents. However, it is only useful if background metabolism is not an issue.^15,16^ Purified enzymes, on the contrary, have the advantage of having more control over parameters, but it leads to smaller reaction volumes due to the high cost of purification and the requirement of stoichiometric addition of cofactors. This can be solved by employing cofactor regeneration systems.^17,18^ Finally, formulations like crude-cell extract, cell-free enzyme production, or immobilized enzymes are other viable options. The optimal formulation strongly depends on the application and the techno-economic-ecologic evaluation of the overall process.^13^

The other field of catalysis is chemocatalysis, which often operates at nonphysiological conditions, such as high pressures, high temperatures, corrosive conditions, and organic solvents. In particular, industrial processes utilizing chemocatalysis are often gas-phase processes, where the catalytic transformation occurs on the solid–gas interface, which belongs to heterogeneous catalysis. However, more specialized and complex transformations are often performed in the presence of homogeneous catalysts, e.g., catalysts that dissolve in the reaction media. Examples of homogeneous catalysts are acid–base catalysts or organometallic coordination compounds. The latter offer the possibility of substrate-specific catalyst tailoring, resulting in unprecedented reaction selectivity and yields. Homogeneous catalysts suffer from the challenges of separation from the product mixture and can be susceptible to degradation under high reaction temperatures (>160 °C). Advantageously, in comparison to heterogeneous catalysts, they require less harsh conditions and are often more selective.

These different types of catalysis are often seen as competitive although their combination is a longstanding goal in academia.^19^ In addition to the benefits of novel reaction pathways and the production of new compounds, integrating chemo- and biocatalysis directly with minimal intermediate stages is advantageous for the overall process by minimizing separation steps. As the discussion surrounding the sustainability of chemical reactions grows, the significance of water-phase chemical catalysis is magnified.^20^ This method allows for the integration of chemo- and biocatalysis within physiological reaction conditions. Moreover, it is feasible to combine all catalysis disciplines in nonphysiological, including nonaqueous environments, as microorganisms and enzymes can adapt to such conditions when appropriately chosen or formulated as described above. The primary objective is to achieve a one-step, one-pot method, which represents the simplest and most convenient process mode for utilizing new substrate streams.^21^ When the chemical step is incorporated within the organism’s metabolic processes, it is viewed as an integral part of the metabolism. This integration allows for the combination of both pathways, facilitating the realization of processes that are either unattainable in nature or minimizing the required genetic modifications.^22,23^ This becomes particularly interesting when renewable carbon sources are utilized as educts. Microorganisms can efficiently convert biomass components, as most of them are evolutionarily adapted to these substrates. C1 compounds are more challenging because there are often no physiological metabolic pathways for C1 molecules, so genetic engineering tools are necessary. Furthermore, physical parameters make it difficult to use them for microorganisms as substrate. For example, especially when gaseous C1 molecules are used, gas–water solubility is a challenge. In addition, C1 molecules, such as methanol, are often toxic to microorganisms. In comparison to microorganisms and enzymes, molecular transition metal catalysts have arisen as potent tools for using CO_2_ and H_2_ as substrates, not only for the production of C1 compounds but also for incorporation into more complex reactions.^24,25^ Consequently, integrating microbial conversions with organometallic catalysts and *in vitro* biocatalysis using enzymes can expand the range of available products derived from renewable sources.

In this review, we present how the different types of catalysts can work in parallel or together to utilize renewable carbon sources such as carbon dioxide (CO_2_), biomass, and polymer waste. Especially products out of CO_2_ and in concatenating catalyzed processes to either expand the product spectrum, minimize process and purification costs, or create value- out of waste streams. The utilization of novel feedstocks in processes with combined bio- and chemocatalysis are researched in the Fuel Science Center, a DFG excellence cluster of the RWTH Aachen University.^26,27^

## 3 Utilization of Renewable Carbon Sources

For decades, the chemical industry relied on fossil feedstocks and used around 500 Mt·a^–1^ in the form of coal, natural gas, and oil for the production of fertilizers, plastics, solvents, detergents, and numerous fine and specialty products.^28^ Additionally, around 3700 Mt·a^–1^ of crude oil is upgraded to various types of fuels, especially gasoline, diesel, and kerosene.^29^ While electrification and the use of hydrogen contribute increasingly to the “decarbonization” of the transport sector, a large part of the applications require the high energy density provided by carbon-based liquid fuels. These application areas as well as the chemical sector are fundamentally relying on carbon feedstocks but need to be “de-fossilized” to meet the global climate challenge.^30^ Renewable carbon can be obtained from the recycling of existing carbon-containing macromolecules, for example, the use of plastic waste as a starting material. Plant biomass is already an important energy carrier and is used for the biotechnological production of chemicals and fuels, but potentials for the expansion of cropland-produced biomass are limited, which is why algae and lignocellulosic biomass are moving to the center of attention.^31^ With currently around 3100 Gt of CO_2_ in the atmosphere, CO_2_ is one of the most abundant carbon sources.^32^ However, to exploit the carbon potential of the atmospheric CO_2_ energy-intensive processes are needed to provide high enough CO_2_ concentrations for downstream processes. Alternatively, CO_2_ can be captured more efficiently at point sources such as coal power plants, waste incineration, or cement factories.^33^

**Figure 2:**
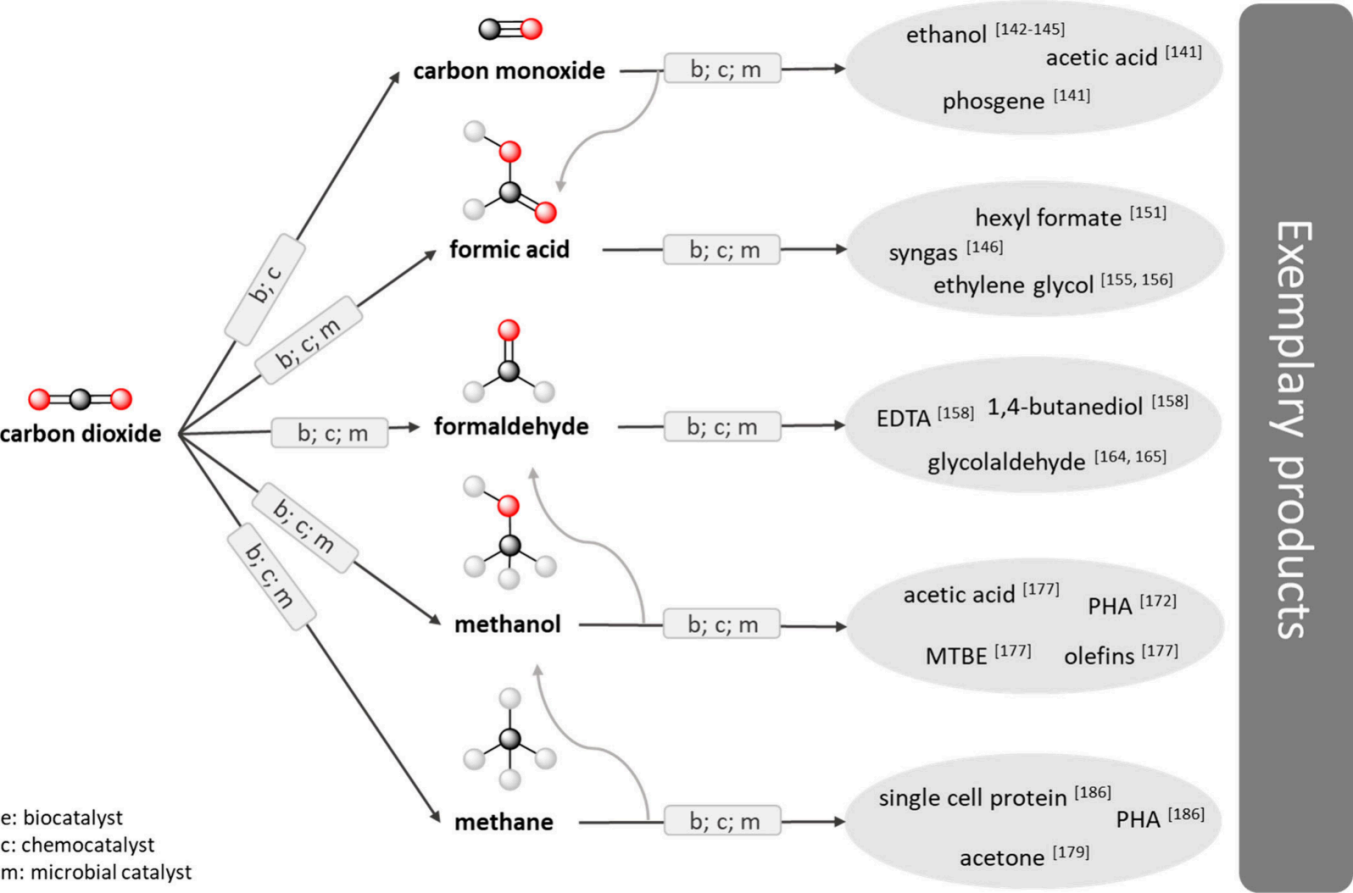
Exemplary product synthesis route starting from CO_2_ with other C1 molecules as intermediates including the different catalyst types. Abbreviations: EDTA, ethylenediaminetetraacetic acid; PHA, polyhydroxyalkanoate; MTBE, methyl-*tert*-butyl ether.

Various conversion processes of these currently untouched renewable carbon sources to platform chemicals and fine chemicals are discussed below, offering multiple routes for each carbon source to show the possibility for concatenation to achieve an optimal process. In Figure 2 examples for product synthesis routes starting from CO_2_ are illustrated, which bases on the current state of research, but does not comprise the whole literature and is continuously growing.

### 3.1 Conversion of CO_2_

CO_2_ conversion by chemocatalysis is mainly focused on the generation of either more reactive C1 building blocks such as carbon monoxide (CO), formic acid, formaldehyde, methanol, or methane, or direct incorporation of CO_2_ into products such as in the formation of (cyclic) carbonates or the formation of linear or cyclic acetals from diols.^34^ Industrially, CO_2_ can be found in reforming, hydrogenation, carboxylation, or mineralization reactions. Biocatalytic CO_2_ conversion offers the possibility to inherently use either existing or specifically designed metabolic pathways to convert CO_2_ to biomass. The most relevant enzyme for natural CO_2_ fixation pathways is ribulose-1,5-bisphosphate carboxylase/oxygenase (RubisCo),^35^ but there are as well other (de)carboxylases that convert CO_2_ in natural cascades to key metabolites and/or in synthetic cascades to fine chemicals.^36^ However, the direct *in vivo* or *in vitro* reduction of CO_2_ to other C1 building blocks is enzymatically challenging and limited. Despite the use of plant biomass for catabolic fermentations, the usage of autotrophic bacteria and microalgae is of increasing relevance. The latter ones have a higher solar-to-biomass efficiency than most plants and are discussed as potent biofuels of the future,^37,38^ but currently commercialized plants only produce <0.1 Mt·a^–1^ of algae biomass.^39^ The intrinsic limitations such as light availability and heat and oxygen removal are difficult if not even impossible to overcome. There are approaches in engineering synthetic CO_2_-fixation pathways for model organisms, such as *E. coli* which could be turned into a hemiautotroph by dividing its metabolism.^40−42^ Although these approaches offer interesting insights into cellular metabolism, they are still on an academic level. Besides photoautotrophs, chemolithoautotrophic bacteria can be used to utilize carbon dioxide as a C-source in combination with H_2_. Conclusive reviews of microbial CO_2_ usage were written by Claasens et al. and by Yang et al.^37,43^

#### 3.1.1 Carbon Monoxide

The primary enzyme to catalyze the reversible conversion of CO_2_ to CO reaction is carbon monoxide dehydrogenase (CODH). Nickel-dependent CODHs, containing nickel and iron in their active site (NiFe-CODH), are frequently studied electrochemically under anaerobic conditions^44−47^ due to their intense electroactivity.^48^ Foundational studies on bioelectrochemical CO_2_/CO interconversions were done by Armstrong and colleagues by showing direct electron transfer with NiFe-CODH adsorbed to a pyrolytic graphite edge electrode.^49^ Electron transfer for the reduction of CO_2_ to CO was demonstrated by using methyl viologen as an electron-transfer mediator from the electrode.^50^ Armstrong and colleagues further showed the photoreduction of CO_2_ to CO under visible light using CODH coupled with CdS nanocrystals and CODH coadsorbed on TiO_2_ nanoparticles with Ag nanoclusters.^51,52^ Electrochemical ways to sustainably produce CO from CO_2_ are mostly performed by heterogeneous electrocatalysts, such as palladium, silver, or gold, which show good activities.^53−56^ Nevertheless, there are also examples of heterogeneous and homogeneous photocatalysts capable of the reduction of carbon dioxide to carbon monoxide with good to excellent selectivities.^57−60^ However, all reactions, if performed in batch reactor setups, suffer from the disadvantageous thermodynamics of the reverse water–gas shift reaction (rWGSR) and require reactor setups that allow continuous CO removal to increase overall yields and efficiencies.

#### 3.1.2 Formate/Formic Acid

Formation of formate from CO_2_ is possible with a variety of different catalysts. The first example with a metal catalyst was reported in 1976 by Inoue^61^ then followed by Leitner^62^ in 1993 performing the reaction in an aqueous medium for the first time. Since then, plenty of different metals and ligands have been used. The most active catalyst was reported by Klankermayer^63^ achieving TONs up to 4.65 · 10^6^ while the most efficient catalysts reach a TOF of 1.1 · 10^6^ h^–1^.^64^ Besides examples using noble metals,^65−71^ there are extensive investigations regarding complexes with earth-abundant central atoms like Ni, Co, Fe, and Mn.^63,67,72−83^ Literature also covers heterogeneous pathways and the electroreduction of CO_2_ to formate since the 1980s describing the development of different generations of electrocatalysts.^84−86^ Biocatalytic, formate is predominantly synthesized from CO_2_ by formate dehydrogenases (FDHs, EC 1.17.1.9) originating from several host organisms such as *Candida boidinii* or *Thiobacillus* sp. KNK65MA, which Amao et al. and Calzadiaz-Ramirez et al. already reviewed.^87,88^ NADH-dependent FDHs have been identified, which are capable of interconverting formate and CO_2_ at the low redox potential of −0.42 V vs standard hydrogen electrode (SHE).^89,90^ For this, various enzyme formulations like whole cell systems^91^ as well as immobilization enzymes were already used.^92−95^ An example of an immobilized system was demonstrated by embedding an FDH from *Candida boidinii* and an NADH in a polydopamine film by copolymerization.^96^ A FDH-assisted microbial electrosynthesis (MES) system was established to produce poly(−3-hydroxybutyrate) (PHB) by feeding formate to a *Cupriavidus necator*, which was engineered for increased CO_2_ uptake.^97^ PHB belongs to the family of polyhydroxyalkanoates (PHA), which are versatile biodegradable biopolymers used as bioplastics for packaging, paper coatings, in the food industry and in the medical field.^98^ One example of PHB’s range of application is tissue engineering and regenerative medicine, as it is (among others) biocompatible, biodegradable and has nonimmunogenicity properties.^99^ As shown above, it can be produced sustainably by microorganisms and is therefore a green polymer. Photoreduction of carbon dioxide to formate was also shown by using bipyridinium salts as an electron carrier necessary for FDH.^100,101^ In recent studies, *Acetobacterium woodii* was converted from an acetogen to a formogen by a genetic deletion and is thus capable of reducing CO_2_ to formate in microbial fermentation.^102^

#### 3.1.3 Formaldehyde

Formaldehyde or formalin belongs to the more challenging products of CO_2_ hydrogenation, due to the high reactivity of the aldehyde group, therefore so far only a few productive chemical reaction systems have been reported. One of the first examples is the photocatalytic hydrogenation described by Inoue in 1979.^103^ More recent investigations are provided by the group of Leitner and Klankermayer which reported the Ru(triphos)(tmm) (triphos = 1,1,1-tris(diphenylphosphinomethyl)ethane; tmm = trimethylenemethane) catalyst in CO_2_ hydrogenation resulting in the formation of dimethoxy methane (DMM).^104^ This catalyst system was further developed and optimized by Trapp^105−107^ while its recyclability was shown by Schaub and Hashmi.^108^ The Klankermayer group expanded on this topic by using Ru(triphos)(tmm) for the synthesis of cyclic and linear acetals from carbon dioxide, molecular hydrogen and diols derived from biomass.^34^ Other groups have used Ni or Co pincer complexes and additives like boranes or silanes to generate formaldehyde equivalents in good yields.^74−76,109^ The enzyme-catalyzed formation of formaldehyde can be achieved by electrolysis of CO_2_-saturated solutions containing methyl viologen (MV^2+^), FDH, and alcohol dehydrogenase (ADH) resulting in a mixture of methanol and formaldehyde.^110^

#### 3.1.4 Methanol

**Figure 3:**
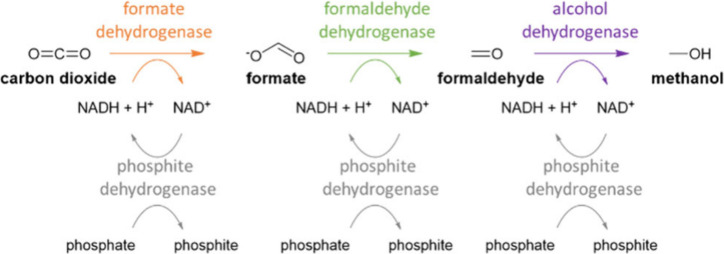
Enzymatic three-step cascade from CO_2_ to methanol including cofactor recycling.

The reduction of CO_2_ to methanol requires six electrons and is therefore considered a difficult reaction. Still, there are plenty of approaches to make this transformation possible. In a metabolically reversed reaction cascade the combination of three NADH-dependent enzymes, formate dehydrogenase (FDH), formaldehyde dehydrogenase (FLDH), and an alcohol dehydrogenase (ADH), enable the production of methanol from CO_2_ (see Figure 3).^111−117^

Another possibility is the electrolysis of a CO_2_-saturated solution containing pyrroloquinoline quinone (PQQ), FDH, and ADH enabling the production of methanol.^110^ Also, a three-enzyme cascade was utilized in a photoelectrochemical cell (PEC) to achieve significant methanol production using solar energy and water, including a cofactor regeneration.^112^ In another approach, enzymes were immobilized using an alginate-silicate hybrid gel matrix on a carbon-felt electrode, which operates without NADH.^118^ Chemocatalytic pathways for the hydrogenation of CO_2_ to methanol have also been investigated using various heterogeneous or homogeneous catalysts.^119−123^ In heterogeneous catalysis catalysts comprised of different metals like Zn, Ag, Cr, and Pd are used. Nevertheless, Cu-based catalysts show the best activities and selectivities.^119^ A heterogeneous catalyst that is at the moment scaled by TotalEnergies is In_2_O_3_ supported on ZrO_2_.^124^ The most prominent pathways in homogeneous catalysis are the direct hydrogenation of CO_2_, which works mostly under acidic conditions, and the indirect hydrogenation of CO_2_, which requires amines to activate CO_2_ via formation of carbarnates in alkaline media.^122^ Additionally, there are some examples of homogeneous catalysts capable of CO_2_ hydrogenation to different products (formate, formaldehyde, methanol), depending on the employed reaction conditions which allow for flexible use.^74−76^

#### 3.1.5 Methane

Common sources of methane are natural gas and biogas plants, which rely on the catabolic conversion of plant biomass.^125^ There are limited approaches for anabolic methane production, such as using microalgae and methanogens. CO_2_ to methane reduction requires eight electrons, prompting a multistep metabolic pathway in nature. Anaerobic archaea can utilize H_2_ and CO_2_ for the production of biomass, with CH_4_ accumulating as a waste stream. But to date, the needed reducing conditions in the fermenter hinder the industrial application of these microbes.^126^ Light-driven reduction of CO_2_ by a genetically modified nitrogenase from *Azotobacter vinelandii* was shown *in vitro* and *in vivo* in a photosynthetic bacterium and *in vitro* by an enzyme.^127^ BES can also be applied to realize electroenzymatic reduction of CO_2_ to methane through a nitrogenase and electron transfer mediated by cobaltocene/cobaltocenium.^128,129^ Methane can also be produced by heterogeneous catalytic CO_2_ hydrogenation, known as the Sabatier process. The power-to-methane concept is undergoing a revival to facilitate long-term space exploration missions by space agencies such as NASA. Since the first description in 1902, many novel catalysts based on Fe and Ni have been developed for low-temperature CO_2_ reduction, with challenges remaining in terms of CO_2_ selectivity and catalytic performance.^130−133^ Homogeneously catalyzed conversion of CO_2_ to methane is scarce because most catalysts lead to CO or formate formation instead of methane as a reaction product.^134^ However, some catalytic systems are reported for the methane formation as shown by Rao et al. by the combination of a photo- and an electrocatalyst.^134,135^

### 3.2 From C1 Feedstocks to Products

As shown above, there are multiple ways to convert CO_2_ into other C1 compounds. The possibilities in converting the selected five C1 feedstocks to end-products either C2 molecules or higher molecular ones are manifold. Various functional groups allow different reactions and various applications as building blocks. There are diverse interaction possibilities of the C1 molecules with the different types of catalysts due to their significantly different properties. On the one hand, these enable a broad product spectrum with diverse applications. On the other hand, there are huge challenges for the integration in the process depending on the characteristics of the C1 components (gaseous or liquid). When considering gaseous C1 molecules, there are particular challenges such as reactor design, transportation or substrate handling and also ensuring that the concentrations and purity are high enough to be reused after e.g. production via gas fermentation.^136^ Nevertheless, liquid components come with their own advantages and disadvantages, and the process must be developed to suit the properties and requirements of the substrates in both cases.

#### 3.2.1 From Carbon Monoxide

CO is one of the important C1 feedstock for carbonylation due to its unique triple bond (two covalent bonds and one coordinate covalent bond) which enables CO to react with a range of radical species and organometallic complexes.^137,138^ This led to the development of industrialized processes that use CO as a feedstock in a mixture of H_2_, such as Fischer–Tropsch, Monsanto, and Cativa processes.^139^ This mixture of CO/H_2_ has been called synthesis gas (syngas), attributed to its use in the industry for the synthesis of methanol and several hydrocarbons. Pure CO can be used for hydrogen production via water gas shift reaction or metal carbonyl production such as Ni(CO)_4_ known as the Mond process.^140^ Other common products are phosgene, acetic acid, formic acid, and methyl formate.^141^ While most of the industrialized processes use metal or organometallic catalysts, bioconversion of CO to valuable products was also shown using anaerobic microorganisms in fermentations, e.g. for organic acids, and alcohol production.^142−144^ The latter is commercialized by Lanzatech for the production of ethanol from steel plant gases.^145^

#### 3.2.2 From Formate and Formic Acid

Formate is stable, nontoxic, nonvolatile, nonflammable, and soluble in both water and organic solvents, making it easy to handle. Formate is the salt of formic acid. By a chemical acid–base reaction it can be converted into formic acid. Formic acid can be used for hydrogen storage due to its 4.35 wt % hydrogen and better transporting properties as well as easier handling compared to gaseous H_2_.^85,86,146^ The application of formic acid in fuel cells is an interesting area for research partly due to the already mentioned advantages over hydrogen and therefore hydrogen cells.^147^ Additionally, chemocatalysts can be used for the decomposition of formic acid into syngas.^146^ As described in the chapter for carbon monoxide, this is the feedstock for industrial production of methanol and acetic acid. In microorganisms, formate is an intermediate in the assimilation pathway of acetogenic bacteria during the growth on CO_2_ or CO, where it can be used to boost biomass production.^148^ Similarly, it can be used to generate NADH by oxidation, improving the biological production of reduced compounds as cofeed by redox balancing.^149^ There are multiple metabolic pathways in natural formatotrophs allowing growth on formate as the sole carbon- and energy source, of which the reductive acetyl-CoA pathway is the most efficient one. Here, only four formate molecules are needed to generate one acetyl-CoA, a universal C2 biomass precursor. Still, there are numerous challenges in using natural formatotrophs, e.g., *Cupriavidus necator*, like limited product spectrum and limited possibilities for genetic engineering. The enzymes used for formate assimilation are quite complex, hindering their integration into a model organism like *E. coli* or *S. cerevisiae*. There are approaches to develop synthetic pathways enabling growth on formate in industrial host strains, but these are far from reaching market maturity.^150^ However, with *in vitro* biocatalysis and isolated enzymes, different product classes could be biocatalytic produced from formate, for example, esters such as hexyl formate^151^ or phenylethyl formate^152^ using lipases or coenzyme-A thioester like formyl-CoA, which is synthesized in an ATP-dependent acetyl-CoA synthase reaction.^153^ Furthermore, formate could be reduced to formaldehyde using a phosphate (Pi)-based route, which is shown *in vivo* and *in vitro* in an enzymatic cascade.^154^ Formyl-CoA is a substrate for further enzymatic upgrading e.g., to ethanol, glycolate, or ethylene glycol.^155,156^ Formate is also used in enzymatic cascades for the cofactor regeneration from NAD^+^ to NADH for the production of various products such as the synthesis of D-mannitol from D-fructose.^157^

#### 3.2.3 From Formaldehyde

Formaldehyde is one of the most versatile platform chemicals. An industrial application is the production of resins by reaction with urea, melamine, or phenol.^158^ It is also used as a C1 building block to form 1,4-butanediol, trimethylolpropane, or neopentyl glycol, which are used for the manufacturing of polyurethane, polyester plastic, and synthetic lubricating oil.^158^ In addition, modern detergents are based on EDTA and NTA, both are produced from formaldehyde. Polymerization of formaldehyde gained more importance in the last decades as polyacetyl plastics are used as light plastics in cars and consumer electronics.^158^ In research, formaldehyde plays an important role in the synthesis of oxymethylene ethers. These compounds are of relevance as they might be a replacement for fossil fuels in the future because of the reduction of NOx and soot emissions.^159,160^ In microbes, formaldehyde is an intermediate in the methanol assimilation pathway, so using formaldehyde directly seems to be an attractive choice. However, it is highly toxic due to unspecific reactions with proteins and nucleic acids, which is why methylotrophic organisms compartmentalize this compound and multiple ways of formaldehyde detoxification exist.^161^ While growth on formaldehyde is theoretically possible, the literature on successful lab tests is very sparse. It is also possible to use enzymes as catalysts. In *in vitro* approaches with enzymes various products are synthesized from formaldehyde Desmons et al. and Germer et al. already summarized.^162,163^ Examples of product classes are aldehydes such as glycolaldehyde,^164,165^ ketones like dihydroxyacetone,^153,166^ carboxylic acid like glycolic acid,^167^ sugars as d-arabinose 3-hexulose 6- phosphate,^168^ or modified amino acids as α-methyl-l-serine.^169^ Formaldehyde is often the starting point in an enzymatic cascade or acts as an intermediate.^153,165^

#### 3.2.4 From Methanol

There are both, yeast and bacteria, which can grow on methanol alone. Although there are different assimilation Pathways, some of the methanol is oxidized and released as CO_2_. While using methanol as feedstock has been a research topic since the oil crises in the 70s, only recent advances in the analysis of methylotrophic microbes and their genetic tools allow a la carte metabolic engineering.^170,171^ Hence, today, the product spectrum is still limited to mostly amino acids, polyhydroxyalkanoate (PHA), and biomass as single cell protein (SCP). Titers for the first two range in the area of 50 to 100 g·L^–1^, while biomass can be produced at up to 28 g_cell-dry-mass-(CDM)_·L^–1^·h^–1^ with titers of up to 250 g·L^–1^.^172^ The production of methylotrophic SCP with *Methylococcus capsulatus* was upscaled to a 250 m^3^ reactor and while this ultimately turned out to be uneconomical, the SCP program accelerated research, and novel approaches in this field will follow. Moreover, methanol is used as a substrate in an enzymatic cascade for starch synthesis with comparable activity as natural starch formation in corn. When the reaction is started with the chemical hydrogenation of CO_2_ to methanol, followed by the enzymatic cascade, even superior activities are achieved.^173^

The use of methanol as the major feed for the chemical industry has been termed as “methanol economy” first by Friedrich Asinger^174^ and later by George Olah.^175^ Both describe in detail how a chemical industry can be built on top of methanol. While in the work of Asinger from the 1980s the discussion focused on coal as a carbon source for methanol, the work of Olah from the 2000s discusses the methanol economy based on carbon dioxide. Some examples of further conversion of methanol can be found in the large scale production of formaldehyde, a transformation, which is also possible in an enzymatic way using methanol dehydrogenases.^176^ Besides this, methanol can be reacted with isobutene to form methyl-*tert*-butyl ether (MTBE), a common octane booster in gasoline, while carbonylation of methanol results in acetic acid, another platform chemical. Processes of lower scale are the formation of methylamines and methyl esters but also hydrocarbons, olefins, and gasoline.^177^

#### 3.2.5 From Methane

In the chemical industry, methane is used in the steam reforming process where it is dehydrogenated to hydrogen with carbon monoxide as a byproduct. This process is of huge importance due to the high demand for hydrogen, especially by the Haber-Bosch process.^178^ Furthermore, methane might be used as a renewable H_2_ storage in a future economy. In research, direct functionalization, dry reforming, and CO_2_-oxidative coupling of methane are among the areas of interest to enable more ways to convert methane into value-added products.^179,180^ Enzymatically, methane can be oxidized to methanol by using methane monooxygenases.^181^ Projects using methane as a carbon source for microbial fermentation suffer from the same problems as those using methanol like limited genetic tools for the organisms. Thus, current efforts are directed toward developing artificial methanotrophs. Initial strides have been demonstrated by heterologous production of the soluble methane monooxygenase (sMMO) in *E. coli*.^182,183^ This work lays the foundation for converting production hosts, such as *E. coli*, into methanotrophs thereby opening up numerous possibilities for various applications.^184^ In many parts of the world methane from fossil resources is the cheapest carbon source (e.g., shale gas in the USA), and hence possibilities for SCP, PHA, and bulk chemicals production prevail, with companies like STRING Biotech or Industrial Microbes demonstrating technology readiness.^184−187^

### 3.3 C2-Compounds

C2-molecules have more binding sites than C1 molecules, which increases the variability. If, for example, we only consider the combination of the five C1 molecules examined here, the synthesis of 15 C2-substrates is already possible. Thus, there are much more C2-substrates than there are C1 compounds. To not inflate this review, only a few are mentioned. Among them, ethanol,^188,189^ glyoxylate,^190^ and acetaldehyde are promising feedstocks for *in vitro* biocatalysis. Exemplary, enzymatic reactions with acetaldehyde are the condensation with formaldehyde using deoxyribose-5-phosphate aldolase (DERA)^176^ or the carboligation of acetaldehyde with benzaldehyde using benzaldehyde lyase from *Pseudomonas putida* (BAL). With the latter, an enzymatic cascade to higher chiral aromatics could be performed, in which sustainable microbial synthesized substrates can be used as well.^191−194^ While industrial model organisms like *S. cerevisiae* can grow efficiently on ethanol, the use of this compound as feedstock has not yet seen any commercial interest. This might be due to the high bioethanol demand and easier alternatives.^195^ Acetate, exactly like formate, can be used to support electron demand in a host producing reduced compounds. Furthermore, acetate can be assimilated via different ATP-dependent enzymes resulting in acetyl-CoA, an important intermediate in many prokaryotic and eukaryotic metabolic pathways.^196^ There is a growing number of molecules that could be produced from this C2 compound like itaconic acid or PHAs, but so far only on a laboratory scale.^197,198^ The challenge of pH changes was used elegantly by Merkel et al. as a feed trigger for itaconic acid production.^199^

### 3.4 Biomass

**Figure 4:**
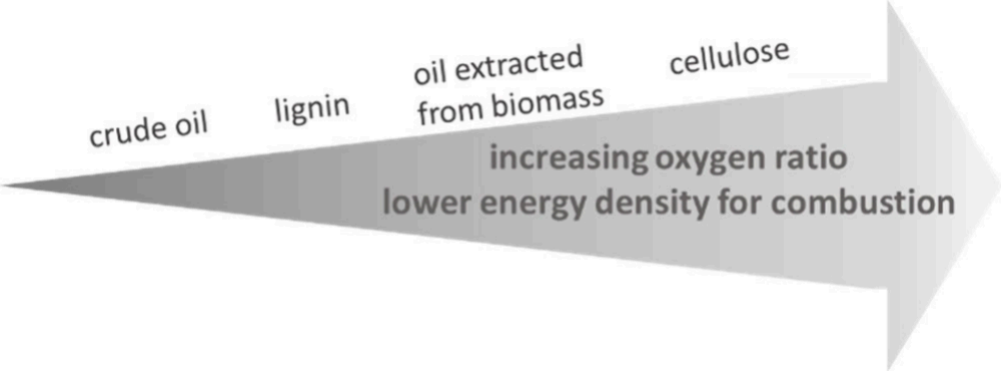
Oxygenation state of fuels versus biomass.

To decrease the dependency on fossil-based carbon sources and to close global carbon cycles, the valorization of renewable biomass presents an interesting opportunity. The oxygenation status is crucial for combustion, for example, as an increase in oxygen enrichment reduces the energy density and therefore the combustion efficiency. An overview of selected fuels and biomass is shown in Figure 4.^200^

**Figure 5:**
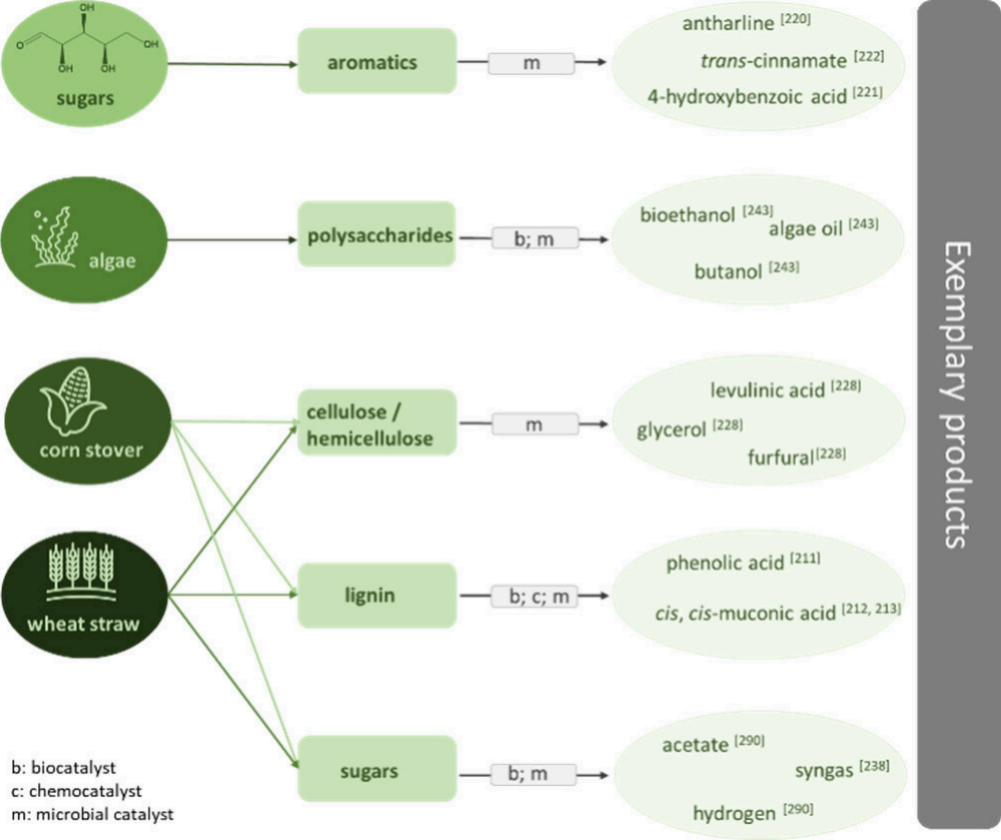
Overview of exemplary biomass-based feedstocks and products.

The International Energy Agency (IEA) Bioenergy has recently published a review on biobased chemicals which provides an overview of the possible products, conversion strategies, and current and expected market sizes.^201^ Herein, selected examples to highlight the variety of conversion strategies from biomass will be discussed (see Figure 5). When using biomass, the first step is to separate lignin from cellulose and hemicellulose using a sustainable method.^202^

#### 3.4.1 Lignin

Lignin is a complex, highly heterogeneous polymer of aromatic monolignols, mainly paracoumaryl alcohol, coniferyl alcohol, and sinapyl alcohol. Lignin occurs in nonedible plants like trees or agricultural residues like wheat straw or corn stover. Although one-step approaches use this complicated feedstock, most projects are based on upstream depolymerization and later upgrading of the monomers.^203−206^ Using biocatalytic processes, enzymes such as lignin peroxidases, manganese peroxidases, laccases, or glutathione-dependent ß-etherase originating from different organisms are often used for lignin degradation.^207−209^ Adding accessory enzymes such as arylsulfotransferase, or peroxiredoxin in the degradation step avoids repolymerization and consequently increases degradation efficiency.^210^ Degradation products are for example aromatic compounds such as phenolic acids, which are further valorized to e.g. biofuels or vanillin.^211^

There are countless lignin-degrading fungi and bacteria, from which fermentations or enzymatic pathways can be utilized, but natural isolates are relatively slow in degrading lignin with most species being ≪1 g·L^–1^·d^–1^.^204,205^ In theory, once the bacteria and fungi can metabolize the monolignols, all compounds in their metabolic network could be produced, but “keeping the ways short”, i.e. upgrading the aromatics to related compounds, improves efficiency. Products with a high research focus are *cis,cis*-muconic acid (MA), an intermediate in lignin catabolization and a precursor of the bulk chemicals adipic and terephthalic acid, nylon, or PHAs. Microbial lignin usage is an increasing field with continuously new technologies, for example, MA can be produced from lignin hydrolysate with a >99% yield.^212,213^

Using chemocatalytic processes, lignin conversion can be achieved with many different catalysts, which have been reviewed in detail in 2018 by Beckham and Sels.^214^ In general, chemocatalytical conversion of lignin can be grouped concerning the catalyst (acid-catalyzed, base-catalyzed, metal-catalyzed).^214−216^ In the acid-catalyzed lignin conversion, diluted mineral acids, such as sulfuric acid, hydrochloric acid, or phosphoric acid are used to break the lignin’s β-O-4 linkage. The same concept can also be applied by using solid acids. In the base-catalyzed lignin conversion, again, the β-O-4 motif is targeted, by forming a quinone methide, which is prone to nucleophilic attack and can be converted to the respective lignin monomers. When discussing the metal-catalyzed lignin conversion, mostly a differentiation between reductive conversions and oxidative conversion can be made. In the reductive conversion of lignin mostly substituted methoxyphenols are formed as intermediates. For the oxidative lignin conversion, often a more complex product spectrum is obtained, since in addition to the cleavage of β-O-4 linkages, also C–C bonds, aromatic rings, and other linkages in lignin can be converted. Mostly, phenolic compounds and carboxylic acids are observed as product classes in the oxidative lignin conversions.^214−216^ The separation of aromatic compounds is still challenging, however, there exist methods for separation as centrifugal partition chromatography (CPC).^217^

#### 3.4.2 Selective Production of Defined Aromatics from Sugars

A major challenge for substituting fossil carbon sources as feedstock for the chemical industry is the limited access to aromatics from renewable carbon sources in an efficient manner. Since the separation of lignin, the major biobased source of aromatics is very challenging, efforts are being made to produce aromatics selectively and in a defined manner using microorganisms. Defined aromatics can be produced directly from sugars by microorganisms. As an example, the conversion of second-generation feedstocks can be catalyzed in metabolic engineered *Pseudomonas taiwanensis*(218) or *Corynebacterium glutamicum*.^219^ In a fermentation with enzymatically engineered *C. glutamicum* 5.9 g/L anthranilate, which is a precursor for polyurethane, was synthesized, from a glucose and xylose mixture.^220^ Other aromatic products from fermentations are, for example, 4-hydroxybenzoic acid,^221^*trans*-cinnamate,^222^ or 4-coumarate.^223,224^

#### 3.4.3 Cellulose and Hemicellulose

Since the transformations from cellulose and hemicellulose into platform chemicals are manifold, herein, only general conversion pathways are mentioned. More detailed information can be found in more narrow reviews elsewhere.^225−228^ Cellulose and hemicellulose are most prominently found as intermediates in the lignocellulosic biorefinery (LCB).^229^ Commonly, cellulose can be hydrolyzed to glucose, from which a variety of different compounds can be produced, both biocatalytically and chemocatalytically.^226,227^ Importantly, platform chemicals originally selected by the Department of Energy (DoE) in 2004 and refined in 2010, can be produced starting from glucose.^230,231^ Moreover, the direct conversion of cellulose to levulinic acid, sugar alcohols, ethylene glycol, and chloromethylfuran can be achieved among others.^225−227,232−237^

Hemicellulose can first be converted to C5 sugars but is most often converted to furfural, which itself presents one of the DoE’s platform chemicals. Notably, while huge efforts were made in the last three decades, only a few commercial ethanol plants are running using wheat straw as a carbon source.

#### 3.4.4 Biomass Gasification

While the use of complex biological material like lignin offers a bypass to the food vs fuel debate, it necessitates sophisticated biomass pretreatment steps and still leaves large parts unused. One way around this problem is biomass gasification to syngas, whose uses are explored above.^238^ Depending on the input, pretreatments such as pelleting, drying, or milling might be required before the biomass is gasified by reactions with steam at >600 °C and >30 bar, which at a steady state produces enough heat to be self-sufficient. Besides the main products CO_2_, CO, and H_2_, the gas contains impurities that need to be removed.^238^ Today, there exist industrial gasification platforms, the largest is in Finland in Vaasa city, in which a circulating fluidized bed gasifier with a capacity of 140 MW is used.^239^

#### 3.4.5 Polysaccharides from Algae

Algae are rich in polysaccharides, which can be enzymatically degraded into monosaccharides, which are being used, e.g., as feedstocks in fermentation.^240,241^ The toolbox of carbohydrate-activating enzymes (CAZymes), containing glycoside hydrolases, glycosyl transferases, polysaccharide lyases, and carbohydrase esterases among others are suitable for polysaccharides degradation.^242^ Bäumgen et al. recently reviewed the enzymatic degradation of the marine polysaccharides carrageenan, laminarin, agar, porphyran, and ulvan and how these polysaccharides could be valorized.^241^

As described above, microalgae can grow with sunlight and CO_2_ alone producing SCP, algae oil, or fine chemicals. But they can also be harvested and used as feedstock. They contain roughly 50% _dry-weight_ carbohydrates in polysaccharides, mostly made up of arabinose, galactose, glucose, rhamnose, and xylose.^243^ Despite macroalgae being cultivated with a volume over 70 Mt·a^–1^, predominantly for food and fodder usage, their application in microbial fermentation is far more challenging, because cell wall polysaccharides contain alginate, mannitol, and laminarin. Here additives are necessary.^244^ Currently, algae biomass as feedstock is explored mainly for commodity chemicals like third-generation methanol, bioethanol, or butanol. However, there is also a growing interest in using them for wastewater treatment and as human and animal food supplements.^243^ One driver is the use of coastal areas that do not interfere with food production and might even be beneficial for maintaining biodiversity.

### 3.5 Plastic Waste

**Figure 6:**
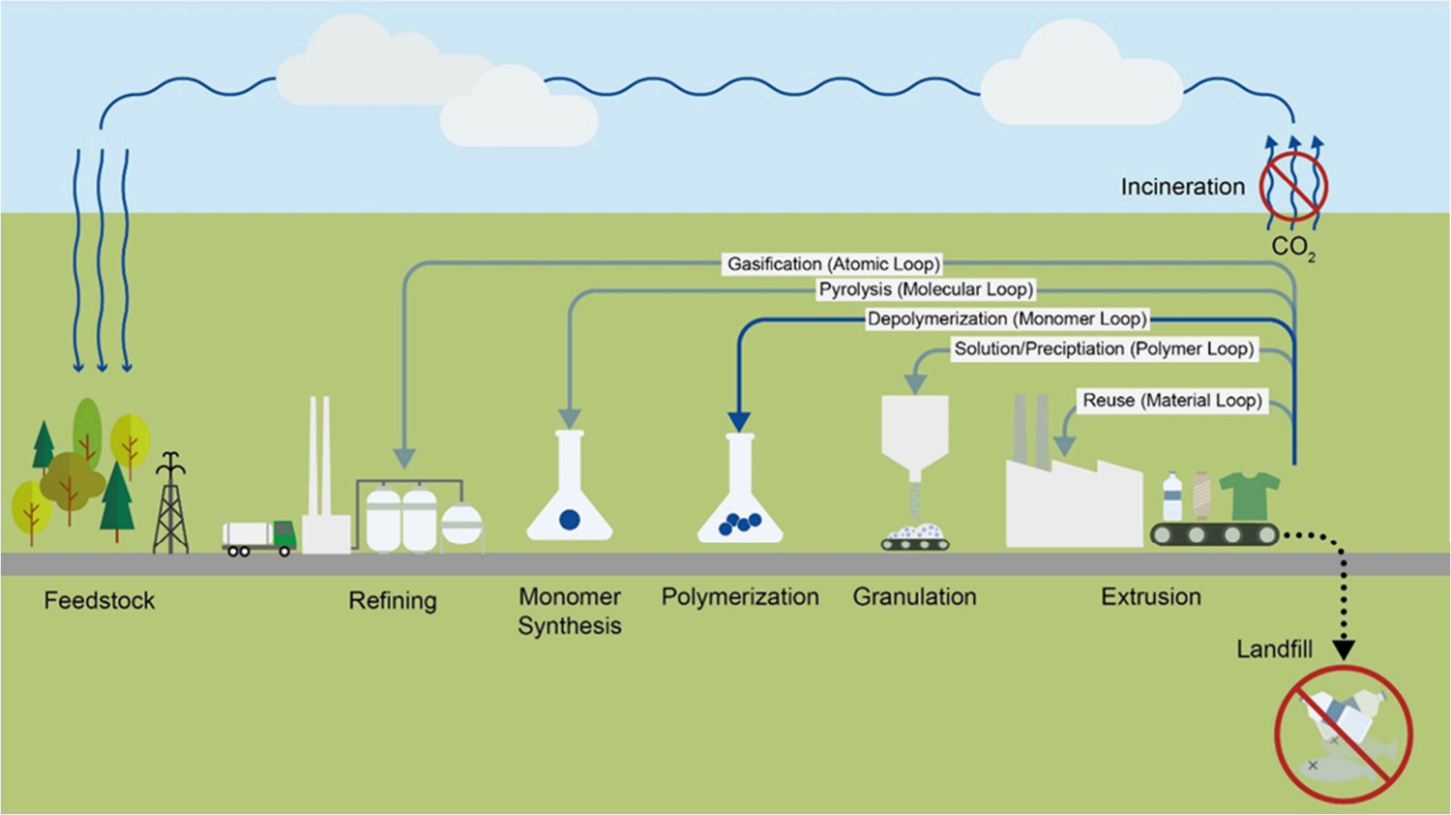
Chemical plastic usage.

Significant potential for use as a renewable carbon source can also be found in the valorization of plastic waste. Similar to biomass, plastics are comprised of a variety of different building blocks. On the one hand, depolymerization of plastics to obtain the monomers for repolymerization can be an option. On the other hand, the conversion of plastics to more versatile platform compounds can be another option. Both strategies are comparable to the biomass conversion, in that most of the already present synthesis effort shall be retained during the conversion of this carbon source. An overview of plastic usage possibilities is illustrated in Figure 6.

Depending on the type of plastic, there are various recycling technologies such as thermal, mechanical, chemical or biological processes. These are explained as examples in this chapter. In order for recycling to be efficient, plastic sorting in particular is crucial, as often only one type of plastic and not a mixture can be used for the recycling process. Sorting can be carried out using fluorescence, electrostatics, infrared, flotation and spectroscopy.^245^

Typically, plastic is first mechanically recycled and then reintroduced into the value chain, e.g., as granules. However, during the mechanical recycling steps, the chain length of the polymers is reduced, so that thermo-physical properties are changed over the life cycles of the recycled plastic and ultimately limit the fraction of recycled material that can be used in new products.^246^ The remaining fractions are sent for chemical recycling to recover chemical building blocks. Various processes such as hydrogenolysis, hydrolysis, solvolysis, and pyrolysis are used in chemical recycling. The difference lies in the driving force and the degree of decomposition of the polymers.^247^ Efficient recycling technologies are required to mitigate the environmental burden of the plastic economy and avoid secondary pollution by the generation of harmful waste streams in the recycling process.^248^ While mechanical recycling is the most energy-efficient recycling technology, chemical recycling has the advantage of higher robustness regarding impurities often found in plastic waste.^249^

Pyrolysis, for example, is a widely used process for recycling plastic waste, in which polymers are decomposed to short-chain hydrocarbons such as olefins and alkanes under high temperature and pressure, with the exclusion of oxygen.^250^ Pyrolysis is therefore a very energy-intensive process that includes the loss of high-value monomer components. Accordingly, chemical recycling processes that maintain the degree of synthesis as far as possible are of great interest for efficient polymer recycling for downstream value creation.^251^

Chemical recycling by depolymerization to monomers followed by monomer purification has the potential to provide recovered monomers and subsequent upgrading with a quality comparable to newly synthesized material. Sing et al. conducted a techno-economic assessment for the chemocatalytic recycling of PET and calculated production costs for purified terephthalic acid sourced from mixed plastic waste of 1.93 $ kg^–1^, which appears to be economically viable. Nonetheless, the eventual feasibility is strongly dependent on local regulations, feedstock prices, and the material efficiency of the recycling process.^252^

Furthermore, chemical recycling paves the way for open-loop recycling by introducing either H_2_ or biomass into the conversion process, yielding products that are of higher value compared to the monomers obtained via depolymerization. For example, hydrogenolysis of consumer PET, PC, and PLA has been demonstrated in the presence of H_2_ and a ruthenium catalyst by Klankermayer et al.^253^ Subsequent life-cycle assessment of this hydrogenolytic polymer conversion revealed the benefits compared to mechanical recycling and energy recovery in cement kilns.^254^

**Figure 7:**
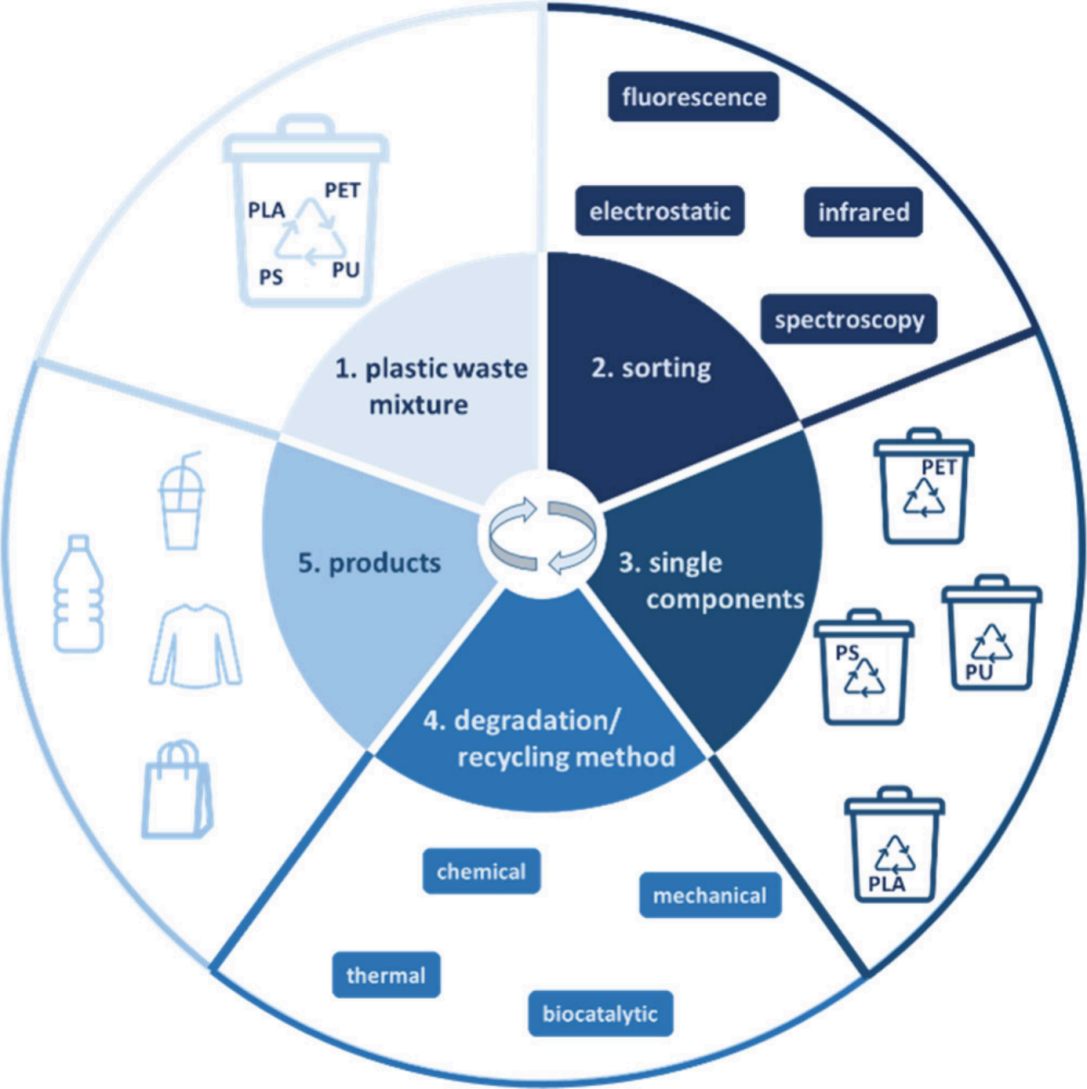
Overview about the circular process from plastic waste for products. Abbreviations: PLA, polylactic acid; PS, polystyrole; PU, polyurethane; PET, polyethylene terephthalate.

Conversion of polymer waste with the integration of biomass to yield cyclic acetals has also been shown for polyoxymethylene (POM) recycling in the presence of biobased diols.^255^ Plastic waste can also be used as an alternative substrate for microbial conversion.^256,257^ Microbial upcycling comes with the interesting feature of incorporating CO_2_ as a final electron acceptor into the metabolic network. This enables new synthetic pathways utilizing plastic waste and CO_2_ as feedstocks to produce value-added products.^258^ As reviewed by Tiso et al. there are multiple examples of microbial utilization and upgrading of plastic hydrolysis monomers. For example, using *Pseudomonas*, PHAs could be produced from terephthalic acid (TA) and styrene at rates of up to 0.1 g·L^–1^·h^–1^. To achieve complete conversion of complex monomer blends, approaches with mixed microbial cultures are promising and shifting into the research focus.^259,260^ Orlando et al. recently reviewed the biocatalytic degradation of plastic waste.^261^ The most studied plastic type for degradation is polyethylene terephthalate (PET), using PETase.^262^ Here Carbios already performed an industrial-scale demonstration plant.^261^ Studies for the challenging degradation of other petroleum-based plastic types like polyurethane (PU) are performed with various enzymes such as esterases,^263^ urethanase,^264^ or laccases.^265^ However, up to now, biocatalytic degradation is not competing with traditional concepts.^261,266^ The circular usage of plastic is shown in Figure 7.

## 4 Integrated Combinations of Bio- and Chemocatalysis

**Figure 8:**
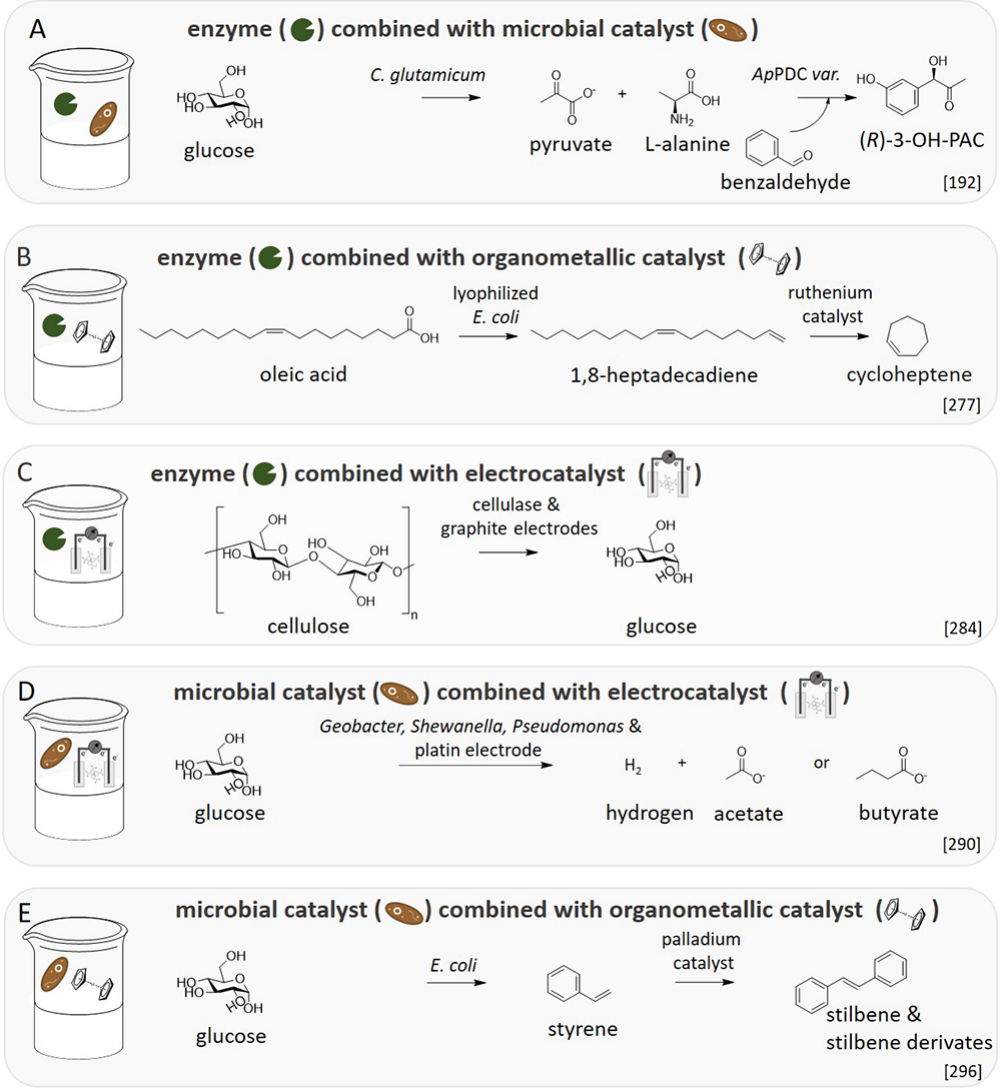
Examples for combining different catalyst types in one-pot processes. (A) Combination of enzyme with microbe for the synthesis of (*R*)-3-hydroxyphenylacetylcarbinol ((*R*)-3-OH-PAC). (B) Combination of enzyme with organometallic catalyst starting from oleic acid to cycloheptene. (C) Combination of enzyme with electrocatalyst to use cellulose. (D) Combination of microbe with electrocatalyst to produce hydrogen and acetate or butyrate. (E) Combination of microbe with organometallic catalyst to produce stilbene and stilbene derivates.

Combining different conversion steps in a one-step, one-pot reaction minimizes reaction times and purification procedures needed. However, the downside is that the reaction conditions need to be conflated to meet the demands of all used catalysts. The greatest challenge lies in the compatibility of reaction conditions, as different catalyst types have distinct requirements, as previously mentioned. Inhibitions, stability issues, and varying physiological parameters such as temperature, pressure, and pH are critical factors. For instance, metals can inhibit enzymes due to their toxicity, and microbial byproducts during growth can hinder chemical catalysts as well as enzymes. Finding the appropriate reaction system is another issue, e.g. whether an organic system, an aqueous system or a two-phase system is compatible with all catalysts. Performing a one-pot process, the key challenge is identifying an operational window that is suitable for all the catalysts involved. In addition, two operating options are conceivable for a one-pot process. Here, a simultaneous addition of both catalysts or a time-dependent sequential addition of the catalysts into one pot are possible. This section includes both operating methods. An overview of the various combination possibilities and selected examples are illustrated in Figure 8.

### 4.1 Combination of *In Vitro* Enzymes and Microbes

The combination of enzymes and microorganisms comprises a one-pot system with both catalysts separately added.^267^ In the simplest case, all the enzymes required for the synthesis can be integrated into the microorganism. However, there are reasons to separate the enzymatic and microbial steps, for example, to avoid side reactions or to precisely adjust the catalyst quantities for optimal flow of substances. In these cases the enzyme is added in another formulation separate to the microbes. Labib et al. utilize *C. glutamicum* to produce pyruvate and alanine. The resultant products were enzymatically converted in a one-pot reaction within the broth with benzaldehyde to produce phenylacetylcarbinol, which was further transformed into metaraminol via enzymatic transamination.^192^ It is essential to separate the transamination step to prevent the enzyme from transaminating numerous side products if present within the microorganism. Moreover, enzymes are used in the preprocessing of lignocellulosic biomass before undergoing microbial transformations.^268^ Another possibility is to coimmobilize microbes and biocatalysts on solids, as described for example by Kiss and co-workers. They developed a system in which living yeast cells are immobilized in alginate gel beads together with a lipase for ethyl oleate synthesis.^269^ A further example is a galacto-oligosaccharide production process in which *S. cerevisiae* and cross-linked ß-galactosidase are also coimmobilized in alginate beads.^270^

### 4.2 Combination of Enzymes and Organometallic Catalysts

The combination of enzymes and organometallic catalysts is an expanding area of research. A prominent example of this combination is the dynamic kinetic resolution of racemic mixtures.^271^ Given the significant potential arising from the combination of whole cells and chemocatalysis, multiple studies are highlighting combinations of these two.^272−276^ A strict categorization is difficult as e.g. some enzymes already contain metals for their physiological activity. Wu et al. for example used lyophilized *E. coli* in an enzymatic cascade for the conversion of oleic acid, which was prolonged by the addition of a Ru catalyst.^277^ Expanding the scope of reaction conditions is also a strong major research focus, for example, demonstrated by the use of deep eutectic solvents to combine a Pd-catalyzed Suzuki C–C-coupling and a subsequent enzymatic transamination,^278^ or by the production of enzyme-metal nanohybrids in protein–polymer conjugates to achieve *in situ* compartmentalization.^279^ Artificial metalloenzymes (ArMs) are a further combination strategy, in which metal cofactors are embedded into protein scaffolds, with which several reactions such as reduction chemistry, C–C bond formation, or oxygen insert chemistry.^280^ An example of combined sequential chemo-enzymatic one-pot catalysis was recently published by Li and co-workers describing the valorization of the C1 molecule formaldehyde. In the first step, acrolein is converted to 3-hydroxypropanal (3-HPA) in chemocatalytic hydration, followed by a biocatalytic conversion in which formaldehyde and 3-HPA are converted to 1,4-dihydroxybutan-2-one catalyzed by BAL variant. These steps could be further coupled with transaminase or carbonyl reductases to produce high-value chemicals for example 1,2,4-butanetriol or 2-aminobutane-1,4-diol.^281^

### 4.3 Combination Enzymes and Electrocatalysts

Using catalytic properties of redox enzymes and electrocatalysis offers advantages in electrolysis and fuel cell applications. Combined with high activity, enzymes also show high selectivity of the substrate, which simplifies fuel cell design.^282,283^ Rezaei et al. showed that cellulases can be immobilized on graphite electrodes coupled with electricity generation.^284^ Also, it has been demonstrated that metals in enzyme active sites are more efficient in catalyzing the production of O_2_ and H_2_ by copper oxidase and hydrogenase, respectively.^285,286^ These applications deal with the scarcity of noble metals used in electrocatalysts.

### 4.4 Combination Microbes and Electrocatalysts

The field of bioelectrochemical systems (BES) is of increasing academic interest. In BES microorganisms are used as catalysts on one or both electrodes of an electrochemical cell. It was shown that certain bacteria can either be used to generate electricity or use electricity to reduce compounds like CO_2_ for microbial utilization.^287^ The underlying mechanism of BES is designed to emulate biological electron transfer through electrodes and electrical energy.^288,289^ Bioelectrocatalysts can operate under mild conditions, are highly efficient and selective, and potentially can use renewable electricity as the only reducing agent.^90^ BES demonstrate efficient conversion of waste-to-energy as microbial fuel cells (MFC) or waste-to-product as microbial electrolysis cell (MEC). As an example, Liu et al. showed bioelectrochemical assisted production of 8–9 mol H_2_/mol glucose which is higher than the current limit of bacterial fermentation production of 4 mol of H_2_/mol glucose.^290^ MFC is known as an approach to harvesting electricity from microorganisms on electrodes as a form of anaerobic respiration. Its application can be used to measure biological oxygen demand and sense arabinose concentrations.^291,292^

### 4.5 Combination Microbes and Organometallic Catalysts

The combination of microbes and organometallic catalysts can serve various purposes, including generating substrates for the microbes, upgrading microbial products, or acting as intermediates to connect different microbial pathways. As an example of the initial challenge, Lee et al. utilized a Ru-complex to deprotect *p*-aminobenzoic acid (PABA) *in situ*, enabling the free acid to serve as a nutrient for an auxotrophic strain of *E. coli* strain.^293^ However, a stoichiometric reaction with the Ru complex cannot be ruled out for conversions that were lower than the amount of catalyst. Additional research has shown the utilization of chemocatalysts to directly produce products that would otherwise be unattainable. The chemocatalyst can be seen as a simplified enzyme that does not require gene integration into the genome. Liu et al. utilized a hemin/Fe(III) complex to restore respiration in *Lactococcus lactis* and increase diacetyl production, a process that conventionally occurs through nonenzymatic decarboxylation.^294^ Moreover, they introduce heterologous genes to convert diacetyl into 2,3-butanediol^294^ or acetoin,^295^ effectively linking two pathways using chemocatalysis. Another example is the *in situ* conversion of styrene produced by *E. coli* to stilbene and stilbene derivatives using a palladium catalyst, as demonstrated by Maaskant et al.,^296^ or to different phenyl cyclopropanes with a Fe(III)phthalocyanine catalyst and ethyl diazoacetate, as shown by Wallace et al.^21^ Rather than upgrading microbial products, Sirasani et al. used H_2_ produced by an engineered *E. coli* as a cofactor to perform Pd-catalyzed hydrogenation.^297^ Stewart et al. used lysine as an organocatalyst, a more biocompatible chemocatalyst instead of otherwise reported transition metal catalysis for the conversion of unsaturated aldehydes from n-aliphatic alcohols, produced by *Gluconobacter oxidans*.^298^ As a completely different approach, microbes such as *Shewanella oneidensis*, *Cupriavidus metallidurans*, or *E. coli* can also be used to aid radical polymerizations of exogenous redox-active metal catalysts such as iron or copper. Fan et al.^299^ and Bennet et al.^300^ used bacteria not only to initiate chain reactions but also to mediate the activity with the tightly controlled redox balance of the cell.

Another reason to combine different types of catalysis might be the utilization of waste streams. Taking the biggest biotechnological example is the production of bioethanol. The pretreatment of feedstocks to fermentable sugars is often carried out chemically, followed by enzymatic or chemical hydrolysis, and finally yeast fermentation.^301^ Bioethanol production is fast with >4 g·L^–1^·h^–1^, efficient with >90% theoretical yield, and the product can accumulate up to 150 g·L^–1^, facilitating production.^301,302^ Nevertheless, even starting from glucose at the maximum theoretical yield of 100%, one-third of the carbon atoms are lost as CO_2_ during the process. Unlike side products such as glycerol,^303^ the microbial generation of CO_2_ as a byproduct is unavoidable due to the necessity of pyruvate decarboxylation for the reaction enthalpy. In 1991, Trost introduced the concept of atom economy with the objective of more precisely characterizing the efficiency of (chemo-)catalysis.^304,305^ For a reaction to be considered effective, it is essential to achieve not only high yields but also to minimize the wastage of “atoms”. To increase the atom economy, especially concerning the valuable carbon of bioethanol fermentations, the scope of biological conversions has been exhausted, but not the catalytic spectrum. The field of organometallic CO_2_ conversions has been the subject of long-standing research, using electricity^306^ or green H_2_.^24^ Additionally, as previously mentioned, catalytic CO_2_/H_2_ conversions have the potential to produce formate (CHOO^–^), formaldehyde (CH_2_O), methanol (CH_3_O), or methane (CH_4_), each presenting distinct challenges and advantages.^24^

In broad terms, microbial biocatalysis and enzyme catalysis are generally acknowledged to exhibit optimal performance within the aqueous reaction milieu, whereas the development of numerous chemical reactions employing transition metal complexes as catalysts primarily occurs in organic solvents. This goal can be attained through the compartmentalization of catalyst types into distinct phases within liquid/liquid multiphase systems,^307^ or by adapting their compatibility to a unified solvent. The latest progress in chemocatalysis within biological environments^308^ and biocatalysis within unconventional media^13^ illustrates the feasibility of determining suitable reaction conditions and combining synthesis with diverse catalysts in a one-pot approach.

**Figure 9:**
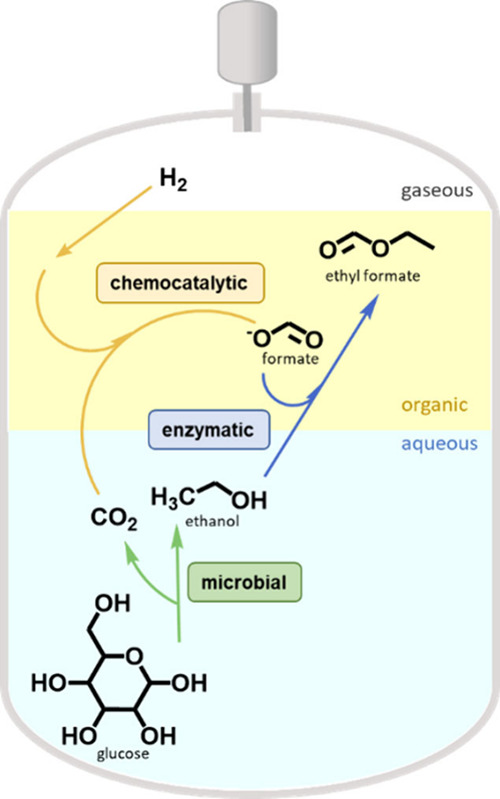
Combining catalysts for improved atom economy in bioethanol fermentations.

Guntermann and Mengers et al., investigated a method to enhance the carbon efficiency of bioethanol production by converting microbial-generated waste CO_2_ through a one-step, one-pot approach (Figure 9).^309^ Formate is nonvolatile, nontoxic and serves not only as a passive “carbon storage” compound but also as a valuable cofeed for both animal^310^ and microbial^311^ applications. Upgrading formate necessitates the coapplication of an organometallic catalyst. Molecular ruthenium complexes are recognized for catalyzing H_2_-driven CO_2_ reduction under mild conditions, which are regulated by the growing cells in one-pot reactions.^312,313^ Separating the catalysts with opposing requirements, either temporally or spatially, simplifies process development, but an integrated system can ultimately outperform complex linked reactors due to fewer separation steps required.^19^ After conflating the reaction conditions, bioethanol fermentation occurred within a high-pressure reactor under 120 bar of H_2_ pressure, concurrently integrated with a Ru-catalyst located in a tetradecane phase for the conversion of CO_2_ and H_2_ into formate. In the end, more than 26% of the initial greenhouse gases were transformed into formate. By separating the fermentation broth from the nonpolar organic phase, efficient recycling of the valuable organometallic catalyst was achieved. Considering a global bioethanol CO_2_ emission of 50 Mt per year, although considerably small compared to the 31,900 Mt per year^314^ from fossil fuels, widespread adoption of this reaction concept could potentially lead to a CO_2_-negative production of 13 Mt per year of formic acid. This concept can be extended further. Both formate and ethanol have the potential to be enzymatically converted into larger, more valuable molecules. Lipases or carboxylic acid reductases (CARs, E.C. 1.2.1.30), can facilitate this conversion process.^315^ CARs typically, utilize ATP to reduce acids and NADPH as a hydride donor. However, in the absence of NADPH, a nucleophile such as ethanol can intercept this reaction, resulting in the formation of esters. With this promiscuous activity, ethyl formate could be generated in the same one-step, one-pot process, leveraging the full range of catalytic potential. The FSC collaborations are currently exploring this pathway.^316^

## 5 Combining Bio- and Chemocatalysis in Reaction Cascades

Although it is (academically) desirable to achieve the complete conversion from renewable carbon source to a product in a one-step, one-pot process, splitting this into a sequence of different catalytic activities increases the degrees of freedom to design the process. As shown for the production and conversion of C1 compounds, microbial, enzymatic, and organometallic catalysis can work in parallel for most of the time. In this way, different catalyst types can be selected and carefully chosen which process conditions should be changed for which intermediate product to minimize cleaning costs. Frequently, academic approaches are initially tested for feasibility on a laboratory-scale with purified glucose instead of preprocessed biomass. The pretreatment of, for example, lignocellulosic biomass is a complex endeavor and an excellent example of the combination of different types of catalysis. While physical pretreatment is fast, chemical pretreatment already hydrolyses some of the polysaccharides, and biological pretreatment has low investment costs due to easy handling.^317,318^

A combination of electrocatalysts and biocatalysts implemented in a continuous-flow setup presents an effective strategy for synthesizing a wide range of fuels and fine chemicals.^319^ This is exemplified by the synergistic integration of various biocatalysts and O_2_-tolerant soluble hydrogenases (SH) from *C. necator* with the electrolysis of water to generate H_2_.^319^ In this approach, SH plays a pivotal role in cofactor regeneration, where it uses the generated H_2_ to reduce nicotinamide cofactors such as NAD^+^. The reduced cofactor is then used by the biocatalyst NADH-dependent imine reductase to produce cyclic amines.^319^ SH also demonstrates remarkable efficacy in regenerating NADPH during the production of various *N*-heterocycles, achieving biocatalytic conversions of up to 99%.^320−322^ Additionally, SH has been shown to recycle flavin cofactors, extending its versatile use to flavin-dependent biocatalysis. This includes the recycling of FMNH_2_ for the selective hydroxylation of alkanes and stereospecific reduction of cyclic enones to respectively produce alcohols and cyclic ketones, as well as FADH_2_ for the asymmetric epoxidation of styrene.^318^ The combination of SH with a diverse set of biocatalysts provides an efficient approach to the hydrogenase-based production of a wide range of acids, amines, and alcohols.^323−325^

**Figure 10:**
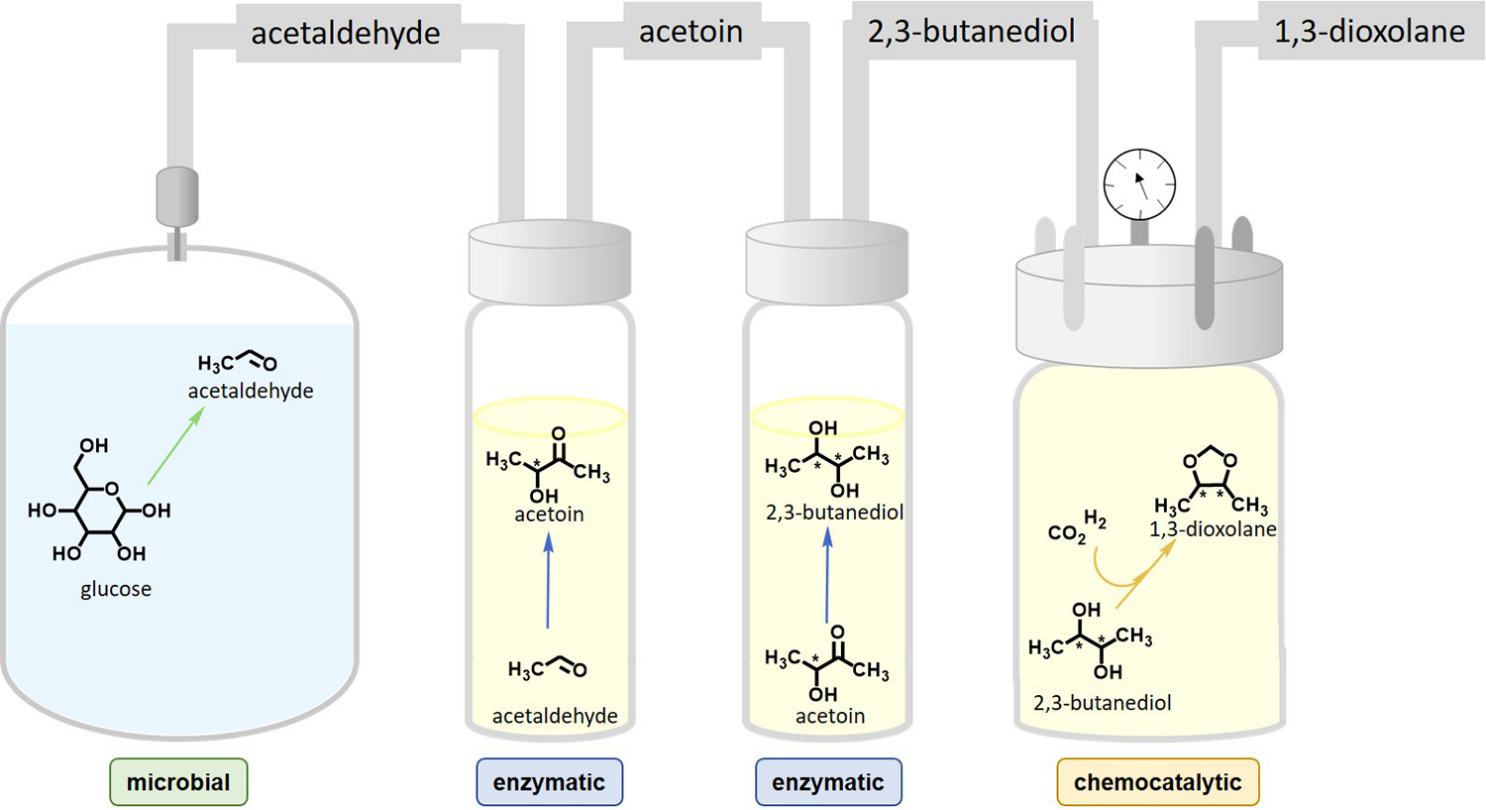
Combining catalysts for novel biohybrid fuel production from biomass.

Interesting candidates for alternative low-carbon, low-emission fuels and “biohybrid″^63,104^ production pathways, including short-chain diols like 1,3-butanediol,^326^ are being investigated within the FSC.^327^ Butanediols can be synthesized directly from biomass using *Enterobacter* or *Klebsiella* strains under anaerobic conditions. Nevertheless, the energy-intensive separation of this hydrophilic, high-boiling compound from an aqueous reaction system through distillation makes this approach unfavorable.^328^ This issue can be circumvented by integrating whole-cell and enzyme catalysis. Mengers et al. suggested a system for yeast-based acetaldehyde production with *in situ* gas stripping and capture.^329^ Because of acetaldehyde’s high vapor pressure, this compound evaporates from the bioreactor, effectively “self-distilling”. Based on this product, Spöring and Graf von Westarp et al., have developed a two-step enzymatic cascade for converting these biobased acetaldehydes into vicinals within a microaqueous (MARS) environment. Since 2,3-butanediol exists as a product within a monophasic organic medium, downstream processing is significantly simplified.^12^ Therefore, through the integration of diverse catalytic approaches, new production pathways can emerge, reducing downstream costs and making green biomanufacturing more attractive. These biofuels can undergo further development by expanding their scope to include CO_2_ and molecules derived from CO_2_.^27^ As detailed previously, the integration of organometallic catalysis with CO_2_ represents a well-established concept in the field.^24^ The conversion of 2,3-butanediol can be accomplished under relatively mild conditions utilizing organometallic Ru-catalysts.^34,330^ This process from biomass to the finished biohybrid fuel 1,3-dioxolane, in turn, covers the entire catalytic spectrum, utilizing the unique capabilities of each catalyst: complex conversion cascades from biomass with whole-cells, organotolerance with high selectivity using enzymes and efficient incorporation of molecular CO_2_ through chemical catalysis (Figure 10).^316^

Furthermore, instead of using the combination of CO_2_ and H_2_, the final reaction step to form the cyclic acetals can also be achieved by switching the feedstock. For one, acetal formation has been shown to progress with formic acid instead of CO_2_ and H_2_.^331^ However, it is also possible to utilize plastic waste, specifically POM, as the source for the methylene group in the acetal, as discussed earlier.^255^ Taken together, the formation of cyclic acetals by the concatenation of different fields of catalysis enables a high degree of flexibility in the underlying renewable carbon feedstock.

When new processes are developed, they must be evaluated in terms of their ecological and economic impact. The process illustrated in Figure 10 has already evaluated. Graf von Westarp et al. systematically evaluated the energy demand of the various theoretically possible process routes to the formation of cyclic acetals, including the separation costs. In addition to the choice of catalyst, the solvent (aqueous or organic) was also a degree of freedom. The elaborated process routes were then compared with a benchmark process and the main cost drivers were identified, both for the reaction characteristics (yield, titer) achieved on a laboratory-scale and for the theoretically possible ones. The added value of such early stage process development lies in the guidance of catalyst development, as bottlenecks can be identified at an early stage and development can be channeled in the right direction. In the given example of producing cyclic acetals, the MARS system was identified as advantageous and the improvement of the stability of enzymatic catalysis (ligation) against inhibiting aldehydes was identified as a decisive lever for further research. These manner of evaluations are relevant for all developed processes. An in-depth economic and ecological evaluation of the process would ideally be supported by a life-cycle assessment^332^ and a detailed cost estimation, which require references and resources and are extremely time-consuming. Thus, it is difficult for body research in this early state. The following metrics can therefore be used for a rough estimate. The “E-Factor”^333^ is a criterion especially estimating environmental impact which considers the actual amount of waste of a process (mass of waste/mass of product). Ideally, it is zero and as higher the factor, the higher the negative environmental impact. Another metric concerning the environmental impact is the climate “C-factor” (C Factor = kg CO_2_ emitted/kg product). This factor represents the overall mass-specific CO_2_ emissions linked to the production of a particular product.^334^ Measurements concerning process efficiency are for instance the yield of the reaction (Y_reaction_=mol_product_/mol_substrate_), the yield of the biocatalyst (Y_catalys_t = g_product_·g_catalyst_^–1^), the final product concentration, and the space-time yield (STY).^335^ The precise process evaluation becomes more critical when a process is transitioned from an academic scale to an industrial one.

As shown above, novel green carbon feedstocks for the production of chemicals can only be effectively implemented through the combination of different types of catalysis. The combination of different types of catalysis can also be found beyond the laboratory-scale. As part of the government funded project Rheticus, a pilot plant operated by Siemens and Evonik is already running in Germany combining the electrochemical conversion of CO_2_ to CO and the subsequent fermentation using *Clostridium* to butanol and hexanol.^336^ As part of the Haru Oni project, Siemens has built the first integrated commercial CO_2_-to-eFuel plant in southern Chile. Energy is generated using a wind turbine to produce green hydrogen and power a direct air capture facility. CO_2_ + H_2_ are converted in a first step to methanol and subsequently to gasoline.^337^ Although this plant currently only uses (electro)chemical conversions, its extension to host fermentative production lines to value chemicals cannot be excluded.

## 6 Future Prospects for Concatenated Catalysis

Taking into account the above-mentioned advantages in a concatenated catalysis process, it is worth considering one-pot approaches, but there might be challenges, which make a two-pot process outperform the one-pot system. Starting from the molecular structure of a given product there are many synthesis routes from renewables possible. Similar to the “retrosynthetic analysis” in organic chemistry, a “retrosynthetic pathway design” can be taken to identify such novel connections between products and feedstocks.^338^ Tools are being developed to facilitate these designs, like Retrobiocats,^339^ but no tool combines all catalyst types available to date. The starting point could be biomass, grown with the sun’s energy or C1 compounds produced from CO_2_ with green hydrogen, through solar power also from the sun’s energy, or a combination of both. In the last years, not only academic research but also industrial cooperations have proven, that novel green feedstocks, embedded in mixed-catalysis value chains, are a central part of the defossilization of the chemical industry. And the examples above show that the catalytic routes can be combined harnessing each catalyst’s advantages. With new *in silico* methods for optimizing microbes,^340^ enzymes,^341^ or organometallic ligands^342^ to specific tasks, conflating the reaction conditions to utilize the complete catalytic spectrum is expected to get easier. With the demand for green chemicals growing,^343^ new and interlaced catalytic routes will emerge in the future. Therefore, the different disciplines in catalysis but also from (bio)chemical engineering and biotechnology need to continue to work in interdisciplinary research teams, rather than tackling research problems individually. Interdisciplinary research teams can come with hurdles as trivial as different terminologies, as plainly as a mismatch in scale (e.g., biotechnological basic research vs chemical engineering) and as complex as understanding the fundamental principles of each discipline. Therefore, a common language is essential and can be taught e.g. by interdisciplinary lecture series.

## Abbreviations

BES: bioelectrochemical systems
MARS: microaqueous reaction systems
RubisCo: ribulose-1,5-bisphosphate carboxylase/oxygenase
CODH: carbon monoxide dehydrogenase
NiFe-CODH: nickel/iron-dependent carbon monoxide dehydrogenase
rWGSR: reverse water–gas shift reaction
FDH: formate dehydrogenase
SHE: standard hydrogen electrode
MES: microbial electrosynthesis
PHB: poly(3-hydroxybutyrate)
Ru(triphos)(tmm): triphos = 1,1,1-tris(diphenylphosphinomethyl)ethane, tmm = trimethylenemethane
DMM: dimethoxy methane
MV^2+^: methyl viologen
ADH: alcohol dehydrogenase
FLDH: formaldehyde dehydrogenase
PQQ: pyrroloquinolinequinone
PEC: photoelectrochemical cell
syngas: synthesis gas
PHA: polyhydroxyalkanoate
SCP: single cell protein
CDM: cell dry mass
MTBE: methyl-*tert*-butyl ether
sMMO: soluble methane monooxygenase
DERA: deoxyribose-5-phosphate aldolase
BAL: benzaldehyde lyase from *Pseudomonas putida*
IEA: International Energy Agency
MA: *cis,cis*-muconic acid
CPC: centrifugal partition chromatography
LCB: lignocellulosic biorefinery
DoE: Department of Energy
CAZymes: carbohydrate-activating enzymes
OECD: Economic Cooperation Development
TA: terephthalic acid
PET: polyethylene terephthalate
PU: polyurethane
PBA: *p*-aminobenzoic acid
MFC: microbial fuel cells
MEC: microbial electrolysis cell
CARs: carboxylic acid reductases
3-HPA: 3-hydroxypropanal
SH: soluble dehydrogenases
STY: space-time-yield
